# Smo-Shh Agonist Purmorphamine Prevents Neurobehavioral and Neurochemical Defects in 8-OH-DPAT-Induced Experimental Model of Obsessive-Compulsive Disorder

**DOI:** 10.3390/brainsci12030342

**Published:** 2022-03-02

**Authors:** Ria Gupta, Sidharth Mehan, Pranshul Sethi, Aradhana Prajapati, Abdulrahman Alshammari, Metab Alharbi, Haneen A. Al-Mazroua, Acharan S. Narula

**Affiliations:** 1 Neuropharmacology Division, Department of Pharmacology, ISF College of Pharmacy, Moga 142001, Punjab, India; ria909@gmail.com (R.G.); pranshulsethiptl@gmail.com (P.S.); aradhanapjt@gmail.com (A.P.); 2 Department of Pharmacology and Toxicology, College of Pharmacy, King Saud University, P.O. Box 2455, Riyadh 11451, Saudi Arabia; abdalshammari@ksu.edu.sa (A.A.); mesalharbi@ksu.edu.sa (M.A.); halmazroua@ksu.edu.sa (H.A.A.-M.); 3 Narula Research, LLC, 107 Boulder Bluff, Chapel Hill, NC 27516, USA; acharannarula@icloud.com

**Keywords:** obsessive-compulsive disorder, 8-OH-DPAT, Smo-Shh, purmorphamine, fluvoxamine, serotonin

## Abstract

Obsessive-compulsive disorder is a mental disorder characterized by repetitive, unwanted thoughts and behavior due to abnormal neuronal corticostriatal-thalamocortical pathway and other neurochemical changes. Purmorphamine is a smoothened-sonic-hedgehog agonist that has a protective effect against many neurological diseases due to its role in maintaining functional connectivity during CNS development and its anti-inflammatory and antioxidant properties. As part of our current research, we investigated the neuroprotective effects of PUR against behavioral and neurochemical changes in 8-hydroxy-2-(di-n-propylamino)-tetralin-induced obsessive-compulsive disorder in rats. Additionally, the effect of PUR was compared with the standard drug for OCD, i.e., fluvoxamine. The intra-dorsal raphe-nucleus injection of 8-OH-DPAT in rats for seven days significantly showed OCD-like repetitive and compulsive behavior along with increased oxidative stress, inflammation, apoptosis, as well as neurotransmitter imbalance. These alterations were dose-dependently attenuated by long-term purmorphamine treatment at 5 mg/kg and 10 mg/kg i.p. In this study, we assessed the level of various neurochemical parameters in different biological samples, including brain homogenate, blood plasma, and CSF, to check the drug’s effect centrally and peripherally. These effects were comparable to the standard oral treatment withfluvoxamine at 10 mg/kg. However, when fluvoxamine was given in combination with purmorphamine, there was a more significant restoration of these alterations than the individualtreatmentswithfluvoxamine and purmorphamine. All the above findings demonstrate that the neuroprotective effect of purmorphamine in OCD can be strong evidence for developing a new therapeutic target for treating and managing OCD.

## 1. Introduction

Obsessive-compulsive disorder (OCD) is marked by recurrent, unusual as well as distressing thoughts (obsession) such as contamination, excessive washing of hands, hygiene, taboo thoughts, etc. Activities are performed to achieverelief from thesethoughts (compulsions), as patients believe that the completion of the ritual is the only preventive measure for the cause [1]. OCD affects 2.3 percent of persons at some point in their lives [2]. Abnormalities in the corticostriatal-thalamocortical (CSTC) circuit and neurochemical changes have been associated with these behavioral changes [3]. In a number of investigations, researchers found that OCD patients had higher levels of dopamine and lower levels of serotonin and growth factors such asBDNF compared to healthy people [4]. Reversing these neurochemical alterations can help treat and manage OCD. OCD is connected to aberrant neurotransmission, as demonstrated by serotonin (5-HT) [5,6], dopamine [6,7] and glutamate [8,9] abnormalities, as well as neuroinflammation [9,10,11] and other neurochemical abnormalities [11,12]. There is evidence that serotonin deficiency is a key cause of OCD, as selective-serotonin-reuptake medications have varying success in treating OCD-like symptoms [13].

8-hydroxy-2-(di-n-propylamino) tetralin (8-OH-DPAT) is a 5-HT1A-receptor activator that induces preservative and obsessive behavior [14]. It is a well-established model for OCD symptoms [15]. The injection of 8-OH-DPAT into the dorsal raphe nucleus (which contains a pool of 5-HT1A receptors) results in OCD-like compulsive and repetitive behavior [16,17]. 8-OH-DPAT also resets the circadian rhythm of the firing rate and alters sleep and waking behaviors in animals [18,19].

For now, selective-serotonin-reuptake inhibitors (SSRI’s) are the first-line therapy for OCD [20]. These are also used to treat depression and anxiety. These block serotonin transporters that are responsible for reuptake from synaptic clefts in neurons. Blocking these transporters increases 5-HT neurotransmission. Shin et al., 2014 found a link between SSRI treatment and improved CSTC functional connectivity [21]. Fluvoxamine, a very effective selective-serotonin-reuptake inhibitor (SSRI), has little or no impact on other monoamine-reuptake pathways. Fluvoxamine is a weak inhibitor of CYP2D6, a moderate inhibitor of CYP2C19 and CYP3A4, and a potent inhibitor of CYP1A2 [22].

In addition to the neurological system, the Shh-signaling pathway has been demonstrated to be critical in developing several organs, including the nervous system [23,24]. Sonic hedgehog (Shh) is the most extensively researched of the three hedgehog homologsthat are known in vertebrates. Depending on the concentration, it can alter several developmental processes by initiating different transcriptional programs and cell types [25]. Shh-dependent transcriptional factors (TF’s) help determine motor, floor plate, and dopaminergic neurons in the embryonic neural tube [26]. It also influences cell differentiation and development, which helps maintain and regulate the CSTC pathway’s neuronal circuit [27]. The sonic-hedgehog (SHH)-signaling pathway is critical for maintaining the integrity of the blood-brain barrier (BBB) and maintaining immunological equilibrium [28].

Severe neuronal-cell-communication disruption, oxidative stress and glutamate excitotoxicity arise from its downregulation [29,30]. In addition, abnormal Smo-Shh signaling has been found in TBI [31], depression [32], autism [33], epilepsy [34], Parkinson’s disease (PD) [35], glioma [36], and medulloblastoma [37]. For its preventive impact against numerous neurological illnesses, the activation of the Smo-Shh-signaling pathway is an important research field.

Purmorphamine (PUR) is a small chemical agonist for Smo receptors that is purine-derivative in nature [38]. It triggers the Smo-Shh-signaling cascade by binding directly to the Smo receptor. Purmorphamine has been shown to influence brain-neuron proliferation and differentiation and initiate osteogenesis in bone tissue [39,40]. The study’s findings by Rahi et al., 2021 demonstrated that PUR exerts a protective effect by attenuating the behavioral abnormalities in propionic-acid (PPA)-induced autistic rats and restoring various neurochemical alterations [41]. Liu et al., 2020 found that PUR has a protective effect by reducing inflammation and synaptic deficits following hypoxic-ischemic injury in neonatal mice [42]. Purmorphamine has also been shown to minimize BBB damage in animal models of infections and ischemic stroke [43]. Other studies have also reported the neuroprotective and regenerative effect of PUR [44]. PUR has also been demonstrated to promote the development of the blood-brain barrier and to protect hippocampal neurons from oxidative-stress-induced cell death [45].

It has been shown that PUR has a protective effect in a variety of disorders, including Parkinson’s disease (PD) [46], ischemic brain injury [47], and autism [41], but it has not been demonstrated to have a protective effect in treating OCD. This study examinedthe impact of PUR onpreventing OCD-related symptoms such as neurological problems and neurobehavioral changes. A further comparison was made between the efficacy of PUR and that of the standard medicine fluvoxamine, both alone and in combination with purmorphamine.

## 2. Materials and Methods

### 2.1. Experimental Animals

We receiveda total of 48 six-month-old adult Wistar rats weighing 250–300 g from the Central Animal House at the ISF College of Pharmacy in Moga, Punjab (eight total groups, each group included six animals: 24 males and 24 females). Polyacrylic cages with a wire mesh top and soft bedding were utilized to hold the animals in the experiment. All animals were kept under conventional husbandry circumstances, which included a 12 h reverse light cycle, food and water (ad libitum), and a temperature of 23 ± 2 °C [33]. According to the guidelines of the Indian government, the experimental protocol was approved by the Institutional Animal Ethics Committee (IAEC) with registration no. 816/PO/ReBiBt/S/04/CPCSEA06/08/2004 and protocol no. ISFCP/IAEC/CPCSEA/Meeting No.28/2020/Protocol No. 465. Before the experiment, the animals were given time to adapt to laboratory conditions.

### 2.2. Chemicals and Drugs

The 8-OH-DPAT was purchased from Merck life sciences, Mumbai, India. PUR was obtained from Cayman Chemicals Cooperation, New Delhi, India. It was dissolved in dimethyl sulfoxide (DMSO), which wasdissolved in water and wasintra-peritoneallyinjected (i.p.) at a dose of 10 mL/kg [48]. FLX was used as a marketed formulation of SUN Pharma Laboratories Ltd., Mumbai, India. FLX was orally administered at a dose of 10 mL/kg after being dissolved in a distilled-water solution of 1 percent (*v*/*v*) Tween-80 [49]. Analytical-grade drug and chemical solutions were employed, and they were all freshly made prior to use [50].

### 2.3. Experimental Protocol Schedule

The experimental protocol lasted 42 days in total. From day 1 to day 7, an intra-dorsal raphe-nucleus injection of 8-OH-DPAT was given, and from day 8 to day 42, the animal grouping continuously received protocol drug PUR, i.p., and standard drug FLX, p.o. Animals were divided into eight groups using random selection. The current study was unblinded, and the experimenter was well-versed in animal care Group1 Vehicle Control; Group2 Sham control; Group3 PUR per se (10 mg/kg, i.p.); Group4 8-OH-DPAT (8 μg/0.5 µL/2 min IDRN) **[OCD]**; Group5 8-OH-DPAT (8 μg/0.5 µL/2 min IDRN)+PUR (5 mg/kg i.p) **[PUR5]**; Group6 8-OH-DPAT (8 μg/0.5 µL/2 min IDRN)+PUR (10 mg/kg i.p) **[PUR10]**; Group7 8-OH-DPAT(8 μg/0.5 µL/2 min IDRN)+FLX (10 mg/kg p.o) **[FLX10]**; Group8 8-OH-DPAT(8 μg/0.5 µL/2 min IDRN)+PUR (10 mg/kg i.p.)+FLX (10 mg/kg p.o) **[PUR10/FLX10]**.

**Figure 1:**
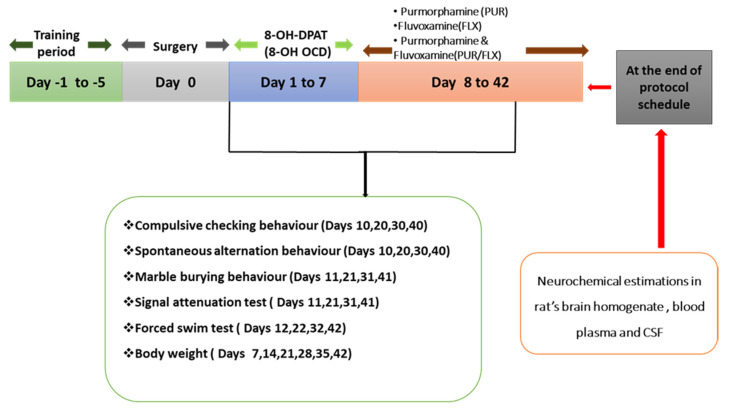
Experiment protocol schedule.

To begin, all of the animals in all of the groups were given the toxin in the morning for 1 to 7 days straight. Following that, from day 8 to day 42, the protocol drug purmorphamine was administered to PUR per se, PUR5, PUR10, and PUR10/FLX10. Fluvoxamine was administered to the animals in the groups FLX10 and PUR10/FLX10, which was the conventional treatment. The PUR10/FLX10 group received the fluvoxamine 30 min after the purmorphamine administration. On particular days, parameters such as spontaneous alternation behavior (SAB), compulsive checking behavior (CCB), marble-burying behavior (MBB), signal-attenuation behavior (SAT), and the forced-swim test (FST) were carried out in order to assess behavioral changes. On the 42nd day of the protocol schedule, all animals that had completed behavioral tests were decapitated, and their brains were separated for biochemical, inflammatory, and neurochemical analyses. An overview of the study’s protocol is summarized in Figure 1.

### 2.4. Experimental Animal Model of 8-OH-DPAT-Induced OCD in Adult Rats

The 8-OH-DPAT-induced experimental model of OCD in the rat was carried out in accordance with the procedure established by Hjorth and Magnusson in 1988.Experimental rats were given intra-dorsal raphe-nucleus injections of 8-OH-DPAT for seven days, and the results were recorded. The study conducted by Hjorth and Magnusson in 1988 clearly demonstrated that 8-OH-DPAT reduced the levels of serotonin in several brain regions related toOCD. Rats were acclimated to laboratory conditions in order to avoid stress and trauma from occurring. Ketamine at a dose of 75 mg/kg was administered to anesthetize the rats (75 mg/kg, i.p.). The anesthetized rat’s body was placed on a warm padded surface with the head secured by incision and ear bars in the stereotaxic frame (Stoelting Co., Wood Dale, IL, USA). It was taken care that the bregma and lambda should be in the same position with proper alignment. Immediately after shaving, the rat’s scalp wascleaned with 70% ethanol to eliminate any debris and fur. A small incision with a scalpel was created in the mid-sagittal plane, the skin was retracted, and the skull was exposed to the bregma and lambda in order to determine the IDRN injection coordinates. Cotton buds were utilized to restrict bleeding, and carboxymethyl-cellulose eye drops containing 0.5 percent *w/v* carboxymethyl cellulose were used to prevent dehydration [51]. 

At anterior/posterior (AP) −7.8 mm, medial/lateral (ML) −3.1 mm and dorsal/ventral (DV) −7.5 mm with respect to the bregma, a hole was created under aseptic conditions and the cannula was implanted [52]. To prevent obstruction of the cerebral aqueduct, the DR cannula was inserted at an angle of 30°. Dental acrylic cement was used to secure the cannula and the surgical incision was then sutured with an absorbable surgical suture and a sterile surgical needle. The cannula was sealed with a detachable plastic ear pin. Gentamicin (50 mg/kg) was given intra-peritoneally to all rats following surgery to prevent sepsis, and lignocaine gel was applied to the sutured area to minimize pain. To prevent bacterial skin infections, Neosporin powder was dusted on the skin and monitored for three days.

In accordance with our newly developed experimental-protocol schedule for OCD, from the first to the seventh day of each week, 8-OH-DPAT (8 g in 0.9 percent saline) was administered through a 10 μL Hamilton microliter syringe attached with a 0.4 mm-outer-diameter hypodermic needle in the dorsal raphe nucleus for 2 min (0.5 μL/2 min). The rats were individually housed in polyacrylic cages with warm fabric and husk during the post-operative period, and careful attention was paid to them until spontaneous movement was restored. A constant temperature of 25 ± 3 °C was maintained in the operating room for approximately 2–3 h following anesthesia induction. After surgery, the animals were kept in their cages for 2–3 days with milk and glucose water to prevent any further physical harm. Their general state of health and dehydration were constantly monitored. Seven days after receiving a proper diet and water intake, the rats began to show symptoms of healing, such as regaining their ability to move freely.

### 2.5. Parameters Assessed

#### Measurement of Body Weight

In the experimental protocol, weekly measurements of body weight were taken on days 1, 7, 14, 21, 28 and 42. Body weight was measured to see if there was a link between compulsive behavior and changes in food intake [53]. 

### 2.6. Behavioral Parameters

#### 2.6.1. Compulsive Checking Behavior (CCB)

Compulsive checking behavior was performed in order to evaluate ritualistic and environment-dependent compulsive behavior. It is characterized by repeated returns to one location in the environment [54]. It was evaluated in a large open field with a dark-colored solid surface to aid in the detection of dark and white objectsvia video. All four small plexiglass boxes (8 cm × 8 cm × 7.5 cm each) remained at the same fixed place in the open field for the duration of the study; two boxes were located at the corners and two boxes were located near the center. For the purposes of locating the animals in the field, the open-field table surface was practically divided into 25 rectangular spaces (locales). The open field and objects were wiped clean after each use by the rats with a diluted solution of an antibacterial cleaner (Lysol).

On the 10th, 20th, 30th, and 40th days of the protocol, each animal was examined for compulsive checking behavior. For ten minutes, the rats were released into an open field with four small plexiglass/glass boxes and allowed to move freely. Virtual cameras captured their every movement. Recorded behavior was used to calculate the frequency of visits and the duration of stops in each locale (the terms“stop” and “visit” are equivalent and interchangeable). The acquired values were utilized to determine the locale with the maximum cumulative visit frequency and the locale with the longest cumulative stop length. The checking behavior was defined in terms of the most-often-visited locale (labeled ‘key place’ or ‘key locale’).

In particular, four distinct types of trackingmetrics were employed to define compulsive behavior in OCD models: (a) checking frequency: total number of visits to the key locale; (b) checking-recurrence time: mean duration of return times to the key locale (‘return time’ is the interval between departure and the next arrival at the locale); (c) stops before returning to the key locale: mean number of places visited between visits to the key locale; (d) checking length: total duration of stay at the key locale divided by the frequency of visits there. These metrics were examined by evaluating video footage of rats moving throughout a ten-minute period [55].

#### 2.6.2. Forced-Swim Test (FST)

On the12th, 22nd, 32nd, and 42nd days, the rats were tested in theFST to determine their depressive behavior. The rats were exposed to the tank for 15 min the first time, during the training session, and 24 h after the initial exposure, the rats were exposed fora second time for 5 min. For the rat, the 6 min testing period consisted of two minutes of habituation and four minutes of the actual test, which yielded the duration of immobility [56]. 

#### 2.6.3. Marble-Burying Behavior (MBB)

Compulsion-like behavior was also measured using the MB test. As part of the protocol, it was performed on days 11, 21, 31, and 41. The rats (*n* = 6 per group) were placed in transparent cages (41 cm × 36 cm × 10 cm) filled with 5 cm of sawdust. On top of the sawdust, in six rows of five, were thirty glass marbles, each measuring 1.5 cm in diameter, that were placed evenly. For 10 min, a 100 W bulb was used to illuminate the test area. After 10 min of exposure to the marbles, the rats were removed and the buried marbles were counted.As long as at least half of amarble wasburied, it was regarded as “buried” [57].

#### 2.6.4. Spontaneous Alternation Behavior (SAB)

The exploratory behavior of rodents, in which mice alternately investigate both arms of a T-maze when both arms are baited with the same reward, provides the basis for spontaneous alternation behavior. It is thought that a decrease or deficit in SAB indicates a preference for the same arm over and over, indicating the compulsive behavior, which is defined by an inability to resist acts that are not helpful [16]. 

It was determined that each rat was examined for spontaneous alternation behavior on days 10, 20, 30, and 40 of the protocol. The T-maze was used in the experiment. The starting and the twogoal arms all measured (50 × 10 × 10). These arms were isolated by wooden doors that were manually opened. Small cups were placed at the corners of each goal arm. On the first day of training (day 1), the animals were given chocolate cake in their cages to help them overcome neophobia of novel food. The next day (day 2), the animals were given 20 min to get used to the T-maze, during which they were permitted to roam the entire space. They were then held in each goal arm for five minutes, at which point the reward was given to them the next day (day 3). The door was lifted, and they could choose either of the goal arms, which were baited with chocolate cake.

A 15 s rest period was given to the animals after they finished eating the food. Cake was placed into an empty cup and the operation was repeated seven times per session. Animals’ decisions (left or right) and the time it took them to reach the target arm (up to 90 s) were recorded. The average number of goal-arm options was then calculated until an alternation occurred. As a result, rats with spontaneous alternation were awarded a score of 1, whereas preservers received a maximum score of 7 [15].

#### 2.6.5. Signal-Attenuation Model

In the signal-attenuation rat model of OCD, rats are trained to exhibit OCD behaviors by pressing a lever for food and then viewing a compound stimulus as a feedback cue. This distinguishes between compulsive and repetitive but not compulsive [58,59].

A lack of response-feedback systems or a lack of signaling that conditions have changed following the organism’s response can lead to obsession and compulsions. As a result, the only way to relax anxious behavior is to successfully complete an activity depicting ritualistic behavior. In OCD, signal attenuation leads to excessive lever pressing. This behavior is called compulsive leverpressing because it may be analogous to the excessive ritualistic and unreasonable behavior seen in OCD. The rats were tested usingthis model on the 11th, 21st, 31st, and 41st days of the protocol schedule.

In our neuroscience lab, Mehan et al. modified and developed the instrument, successfully established and validated it with rodent behavior, and named it a rodent neurobehavioral analyzer for obsessive-compulsive disorder (OCD) and psychiatric disorders. The apparatus consisted of one operant chamber (45 cm × 26 cm × 27 cm), consisting of one lever (2 cm wide) and a food magazine for food delivery withdimensions (5.5 cm × 5.5 cm). Both the lever and food magazine werepositioned at 3 cm from each side of the wall with an inter-distance of 7 cm. A house light of 4 Watts was attached to the operant chamber. Signal was given in the form of light and sound. On providing thesignal magazine, the light was manually turned on along with the sound signal.

Before the experiment began, the rats were handled for around two minutes a day for five days prior to the start of the trial. Handling and behavioral testing were both preceded by a 22 h food-restriction program. Immediately following the session, food was given to the rats in theircages. In the last three days, a tray containing 20–30 food pellets used in operant training was placed inside the cage and then removed. After at least two pellets were eaten by each rat, the tray was removed from the cage. 

Post-training: 

Day 1: Habituation

Rats were trained to collect food pellets from the magazine in the operant chamber. A magazine containing 4–5 food pellets was placed in front of the rats, who were given 5 min to consume all of the pellets from the magazine.

Day 2–3: Magazine training

Rats were trained to collect food pellets from a food magazine. In each trial, a single food pellet was dropped into a food magazine through a food-dispenser tube simultaneous with the onset of the magazine light and sound. The stimulus was turned off after the rat head entered the food magazine or after 15 s with 30 s intervals between each trial. The number of completed trials (magazine entry during stimulus presentation) was observed within 30 min. 

Day 4–5: Lever-press training

In this training session, the delivery of food and the onset of the magazine light and sound were only initiatedwhen the rat pressed the lever. The stimulus was turned off after the rat head entered the food magazine or after 15 s from the lever press had elapsed. During the whole session (maximum 30 min) the rat should have completed 30 trials. 

Day 6: Signal attenuation

In this training, rats were exposed to a stimulus as in the lever-press training, but no food was delivered to the food magazine uponpressing the lever. The rat was trained until it had completed no more than 14 collected trials. 

Test day: 

The test was the same as that of the signal-attenuation stage. The number of completed excessive-lever-press trials were observed and recorded. The obsessive-compulsive-induced experimental rats showed more repeated trials than the normal and treated groups [60].

### 2.7. Neurochemical Alterations Evaluation and Collection and Preparation of Biological Sample

#### 2.7.1. Brain-Homogenate Preparation

After decapitation, brains were extracted and cleaned with ice-cold isotonic saline solution on the 43rd day of the protocol schedule. Ten times (*w*/*v*) ice-cold 0.1 M phosphate buffer (7.4) was used to homogenize the brain samples. Aliquots of the supernatant from the centrifuged homogenate were utilized for biochemical estimations after centrifugation at 10,000× *g* for 15 min [61,62].

#### 2.7.2. Blood Plasma Collection

A sample of blood from the orbital sinus was taken before euthanasia occurred on day 43 of the treatment. Chloroform was used to anesthetize the rats (CHCl3). The animal was then scruffled with the thumb and forefinger of the non-dominant hand, and the skin around the eye was pulled tight. The medial canthus of the eye was punctured, and a capillary was introduced (30-degree angle to the nose). EDTA-containing Eppendorf tubes were used to collect the blood after puncturing the plexus or sinus. The plasma was then separated by centrifugation at 10,000× *g* for 5 min at 4 °C [63]. 

#### 2.7.3. CSF Collection

On day 43, the animals were sacrificedwith CHCl3, and CSF was collected according to the Rosenling et al., 2010 procedure. The rats’ heads were held in place using a device to display the arachnoid membrane. A small incision was madein the musculus trapezius pars descendens, followed by a horizontal incision. A maximum volume of 100 µL CSF was collected by directly inserting a 30 G butterfly-syringe needle into the cisterna magna via the arachnoid membrane. Each sample was centrifuged at a force of 2000× *g* for 10 min at 4 °C within 20 min of being collected. The supernatant was centrifuged and kept at −80 °C until further investigation [64].

#### 2.7.4. Measurement of Smo-Shh levels

Smo-Shh levels in the rats’ brain-homogenate supernatant, blood plasma, and CSF were determined using an ELISA kit from Elabsciences in China. It is important to note that the data arereported as nM/g protein (brain homogenate) and ng/mL, respectively (blood plasma and CSF) [41].

### 2.8. Measurement of Apoptotic Markers

#### 2.8.1. Measurement of Caspase-3

Caspase-3 levels were determined in both the brain homogenate and the blood plasma using an ELISA kit (Elabsciences, Wuhan, China). The process was carried out in accordance with the manufacturer’s instructions. The competitive-enzyme-immunoassay technique was used in this assay, and the results arerepresented as ng/mg protein (brain homogenate) and ng/mL of sample (blood plasma) [65].

#### 2.8.2. Measurement of Bax and Bcl-2 Level

The levels of Bcl-2 protein and Bax protein in the brain homogenate were determined using an ELISA kit (Elabsciences, China) onthe samples. The process was carried out in accordance with the manufacturer’s specifications and instructions. The values arerepresented as ng/mg protein (brain homogenate) and ng/mL (blood plasma) [66]. 

### 2.9. Evaluation of Neuroinflammatory Cytokines

#### 2.9.1. Estimation of TNF-α Level

Using a diagnostic kit from Krishgen Diagnostics, India, the amount of TNF-α in the rats’ brain homogenate and blood plasma was determined in the experiments. All of the samples and reagents were produced in accordance with the instructions provided with the kit. The reaction mixture’s optical density was measured at 450 nmin microtiter plates. The data arepresented as pg/mg protein (brain homogenate), ng/mL, and ng/mg protein (brain homogenate) (blood plasma) [67,68]. 

#### 2.9.2. Estimation of IL-1β Levels

A diagnostic kit from Krishgen Diagnostics, India, was used to test the concentration of IL-1β in the rats’ brain homogenate and blood plasma. According to the instructions on the kit, both samples and reagents were prepared as described. At a wavelength of 450 nm, the microtiter-plate reaction mixture’s optical density was measured. The data arepresented as pg/mg protein (brain homogenate), ng/mL, and ng/mg protein (brain homogenate) (blood plasma) [69].

### 2.10. Evaluation of Neurotransmitters

#### 2.10.1. Serotonin Levels

An electrochemical detector was used to measure the concentration of serotonin in the rats’ brain homogenate. There were two components to the mobile phase: buffer (sodium citrate) and acetonitrile (ACN) in the ratio of 87:13, *v*/*v* (pH 4.5). Smooth separation wasensured by running the sample through the apparatus at a rate of 1 mL/min, and results are expressed in ng/mg protein [70]. 

#### 2.10.2. Glutamate Levels

It was possible to measure glutamate levels after derivatization with the compounds o-phthaldehyde/β-mercaptoethanol (OPA/β-ME). A quantitative examination of the tissue sample was carried out using an electrochemical detector (ECD) and high-performance liquid chromatography by HPLC-ECD Waters, USA, with the results represented as ng/mg protein [71].

#### 2.10.3. Dopamine (DOPA) Levels

High-performance liquid chromatography (HPLC) ECD Waters, USA and an electrochemical detector (ECD) were used to detect dopamine levels in the rat brain. Sodium citrate (pH 4.5) and acetonitrile (87:13, *v*/*v*) were used to buffer the mobile phase. NaH_2_HPO_4_ (NaH_2_HPO_4_) wasused in the buffer, which contained 10 mM citric acid, as well as 25 mM of EDTA (ethylene diamine tetraacetic acid). A voltage of +0.75 V and a sensitivity of 5–50 nA were used in the experiment. A flow rate of 0.8 mL/min was used to accomplish the separation. The samples (20 μL) were manually injected. A concentration of 0.2 M perchloric acid was used to homogenize rat-brain samples and centrifuge them for 5 min at 12,000× *g*. Results are expressed as ng/mg protein [72].

### 2.11. Evaluation of Oxidative-Stress Markers

#### 2.11.1. Acetyl Cholinesterase (AChE) Levels

AUV spectrophotometer was used to determine the quantitative activity of acetylcholinesterase in the rat brain (Pharmaspec, Shimadzu, St. Lenexa, KS, USA). The assay mixture contained 0.05 mL supernatant, 3 mL 0.01 M sodium phosphate buffer of pH 8, 0.10 mL iodide acetylthiocholine, and 0.10 mL DTNB (Ellman reagent). Spectrophotometrically, the absorbance was immediately measured at 412 nm. The enzymatic activity of the supernatant is expressed as protein μM/mg [73]. 

#### 2.11.2. Lactate Dehydrogenase (LDH) Levels

The lactate-dehydrogenase levelin the rats’ brain homogenate was measured using a diagnostic kit from Coral diagnostics, India, and is expressed as unit/mg protein [74].

#### 2.11.3. Superoxide Dismutase (SOD) Levels

The spectrophotometric auto-oxidation of epinephrine was used to evaluate the SOD activity at pH 10.4. The reaction was started by adding 0.02 mL of epinephrine to the 0.2 mL of supernatant in the 0.8 mL of 50 mM glycine buffer. A 5 min spectrophotometric measurement of absorbance at 480 nm was performed. SOD activity is expressed asμM/mg protein [75]. 

#### 2.11.4. Reduced-Glutathione (GSH) Levels

For the purpose of determining the concentration of reduced brain glutathione, 1 mL of supernatant was precipitated with 1 mL of 4% sulfosalicylic acid and cold digested at 4 °C for one hour. They were centrifuged at 1200× *g* for 15 min. Finally, 1 mL supernatant was added to 2.7 mL of phosphate buffer (0.1 M, pH 8) and 0.02 mL of 5.5′ dithiobis-(2-nitrobenzoic acid). The 412 nm wavelength of a spectrophotometer was used to measure the yellow color’s intensity as soon as it was formed. In the supernatant, the glutathione content isexpressed asμM/mg protein [67]. 

#### 2.11.5. Nitrite Levels

A colorimetric assay with Greiss reagent (0.1 percent N-(1-naphthyl) ethylenediamine dihydrochloride, 1-percent sulfanilamide, and 2.5% phosphoric acid) measures the accumulation of nitrite in the supernatant as a sign of nitric-oxide (NO) generation. Supernatant and Greiss reagents of equal volume were mixed together, and the combination was left to incubate in the dark at room temperature for 10 min and then spectrophotometrically tested for absorbance at 540 nm. The supernatant’s nitrite concentration was measured using the standard sodium-nitrite curve and is expressed as μM/mg protein [76].

#### 2.11.6. Malondialdehyde (MDA) Levels

The level of malondialdehyde determines the lipid peroxidation in the rats’ brain homogenate. The amount of MDA wasmeasured by a spectrophotometer after its reaction with thiobarbituric acid at 532 nm. MDA concentration is expressed as nM/mg protein [77]. 

### 2.12. Statistical Analysis

Two-way ANOVA with Bonferroni post-hoc test and one-way ANOVA repeated measures with Tukey’s multi-comparison test were used to evaluate the data. It was determined that *p* < 0.01 was statistically significant. The sample size was estimated after the data wereconfirmed to be normalized and the normality distribution was checked using the Kolmogorov–Smirnov test. GraphPad Prism version 5.03 for Windows was used to generate all statistical results (GraphPad Software, San Diego, CA, USA). The mean and standard error of the mean areused to express the statistical data (SEM).

## 3. Results

### 3.1. Effect of Purmorphamineon Weight Variations in 8-OH OCD

#### Improvement in Body Weight after Purmorphamine Treatment

**Figure 2:**
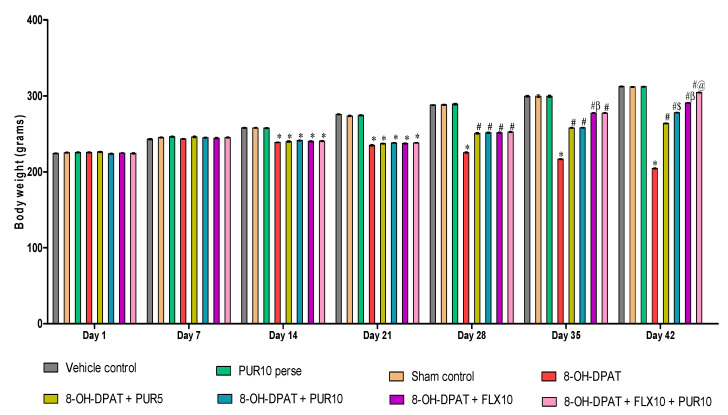
Effect of purmorphamine on body weight in 8-OH OCD. Statistical analysis followed by two-way ANOVA (post-hoc Bonferroni’s test). Values expressed as mean ± SEM (*n* = 6 rats per group). * *p* < 0.01 v/s vehicle control, sham control and PUR10 per se; # *p* < 0.01 v/s 8-OH-DPAT; #$ *p* < 0.01 v/s 8-OH-DPAT + PUR5; #β *p* < 0.01 v/s 8-OH-DPAT + PUR5 and 8-OH-DPAT + PUR10; #@ *p* < 0.01 v/s 8-OH-DPAT + FLX10.

The body weight was measured weekly, i.e., at days 1, 7, 14, 21, 28, 35, and 42 of the protocol schedule. Figure 2 shows the changes in body weight induced by the toxin 8-OH-DPAT compared to the treatment drugs throughout the protocol schedule. The administration of 8-OH-DPAT for the first seven days resulted in a gradual decrease in body weight compared with the vehicle-, sham-, and PUR10-per se-treated groups. Chronic oral treatment withPUR and FLX from day 8 to day 42 showed a gradual improvement in the body weight of animals due to improvements in obsessive behaviors such as less anxiety, fear, stress, and more food intake.

The FLX10-treated rats showed a more considerable improvement in body weight [two-way ANOVA: F(42, 240) = 369.9, *p* < 0.01)when compared with the PUR5- and PUR10-treated rats. Additionally, FLX10 along with PUR10 showed a substantial restoration of body weight as compared to other drug-treatment groups such as PUR5, PUR10, and FLX10. PUR10 was shown to be more efficient in restoring the 8-OH-DPAT-induced reduction in body weight than PUR5, indicating the dose-dependent effect of PUR on restoring the body weight.

### 3.2. Effect of Purmorphaminein the Treatment of Neurobehavioral Abnormalities in 8-OH-DPAT-Induced Experimental Model of OCD in Adult Rats

#### 3.2.1. Improved Compulsive Checking Behavior in the Open Field after Purmorphamine Treatment

This parameter was testedon the protocol schedule’s 10th, 20th, 30th, and 40th days. Normal rats showed checking behavior, i.e., returning to a key locale when exploring. Adult rats treated with 8-OH-DPAT for seven days showed compulsive checking behavior. The rodents visited the key locale the most often compared to other locales, similar to compulsive checking in OCD patients. From day 8 to 42, the therapy medications (PUR and FLX) reduced compulsive checking at the key locale. The following four criteria were used to define the changes in behavior of all treatment groups.

**a.**  **Decrease frequency of checking after purmorphamine treatment**

**Figure 3:**
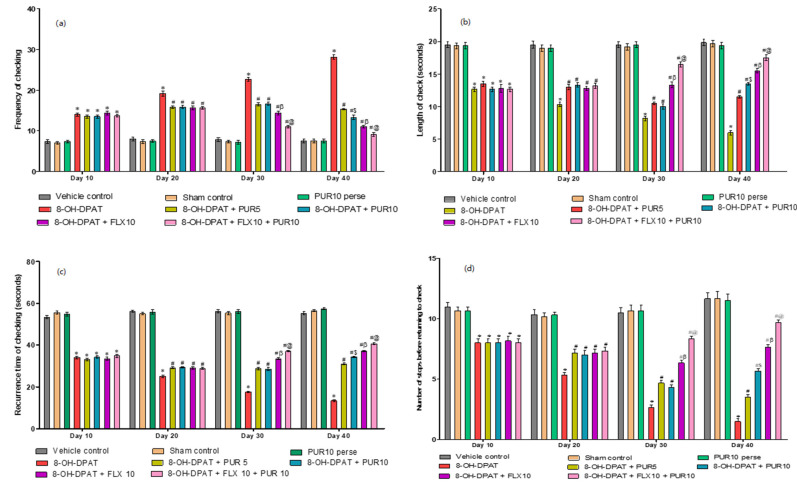
(**a**) Effect of purmorphamine on frequency of checking in 8-OH OCD. Statistical analysis followed by two-way ANOVA (post-hoc Bonferroni’s test). Values expressed as mean ± SEM (*n* = 6 rats per group). (**b**) Effect of purmorphamine on length of check in 8-OH OCD. Statistical analysis followed by two-way ANOVA (post-hoc Bonferroni’s test). (**c**) Effect of purmorphamine on recurrence time of checking in 8-OH OCD. Statistical analysis followed by two-way ANOVA (post-hoc Bonferroni’s test). (**d**) Effect of purmorphamine on number of stops before returning to key locale in 8-OH OCD. Statistical analysis followed by two-way ANOVA (post-hoc Bonferroni’s test). * *p* < 0.01 v/s vehicle control, sham control and PUR10 per se; # *p* < 0.01 v/s 8-OH-DPAT; #$ *p* < 0.01 v/s 8-OH-DPAT + PUR5; #β *p* < 0.01 v/s 8-OH-DPAT + PUR5 and 8-OH-DPAT + PUR10; #@ *p* < 0.01 v/s 8-OH-DPAT + FLX10.

Figure 3a indicates the number of times the rats visited the key locale. The 8-OH-DPAT-treated rats returned to the key locale more frequently than the vehicle-control, sham-control, and PUR10-per se groups. However, by the 20th day of the protocol schedule after drug treatment, the frequency of returns to the key locale had begun to decrease compared to the primarily 8-OH-DPAT-induced experimental model of OCD in adult rats. After 30 days, the FLX10- and FLX10-and-PUR10-treated groups had a significantly lower number of excessive returns to the key locale (two-way ANOVA: F(21,120) = 37.58, *p* < 0.01) than the PUR5- and PUR10-treated groups. Additionally, FLX10 and PUR10 had a more significant effect in reducing excessive returns to the key locale than the treatment withFLX10 alone. After 40 days of PUR10, the treated rats demonstrated more significant attenuation of an excessive number of visits when compared to PUR5 treatment of 8-OH OCD.

**b.**  **Increased length of the check after purmorphamine treatment**

The length of the check was defined as the mean time (in seconds) per visit that the rats stayed atthe key locale or spent time atthe key locale. Compared to the vehicle-control, sham-control, and PUR10-per se groups, the 8-OH-DPAT-treated rats spent less time at thekey locale. However, on the 20th day of the protocol schedule following drug treatment, the length of stay at the key locale began to rise compared with only the 8-OH-DPAT-induced experimental model of OCD in adult rats. At 30 days, the FLX10- and FLX10-and-PUR10-treated rat groups showed a significant increase in time spent at the key locale (two-way ANOVA: F(21,120) = 15.59, *p* < 0.01) as compared to the PUR5- and PUR10-treated rats. Additionally, FLX10 and PUR10 had a more significant effect onincreasing the time that rats stayed at the key locale compared to the treatment withFLX10 alone. After 40 days, the PUR10-treatment group had a significantly increased length of check compared to the PUR5-treatment groupin 8-OH OCD (Figure 3b).

**c.**  **Increase recurrence time of checking after purmorphamine treatment**

Figure 3c depicts the mean time taken by the rats to return to the key locale. The 8-OH-DPAT-treated rats took less time to return to the key locale than the vehicle-control, sham-control, and PUR10-per se groups, indicating the rat’s inability to resist key-locale checking. However, on the 20thday of the protocol schedule following drug treatment, the recurrence time of checking continued to increase compared to 8-OH OCD, i.e., they tookmore time to return to the key locale after investigating the other locales. At 30 days, the FLX10- and FLX10-and-PUR10-treated rat groups took longer to return to the key locale (two-way ANOVA: F(21,120) = 45.78, *p* < 0.01) than the PUR5- and PUR10-treated groups. Additionally, FLX10 and PUR10 had a more significant effect onincreasing the time taken by the rat to return to the key locale compared to the treatment with FLX10 alone. After 40 days of PUR10 therapy, the rats spent more time at other locales and had a prolonged recurrence time of checking compared to PUR5 treatment of8-OH OCD.

**d.**  **Increase the number of stops before returning to key locale after purmorphamine treatment**

The number of stops before returning to the key locale was higher in the 8-OH-DPAT-treated rats compared to the vehicle-control, sham-control, and PUR10-per se groups. However, on the 20thday of the protocol schedule after drug treatment, the number of stops before returning to the key locale started to increase in comparison to the 8-OH-DPAT-treated experimental model of OCD in adult rats. On the 30th day, the FLX10- and FLX10-and-PUR10-treated groups had a relatively higher number of stops before returning to the key locale (two-way ANOVA: F(21,120) = 17.22, *p* < 0.01) than the PUR5- and PUR10-treated rats. Additionally, FLX10 and PUR10 had a more significant effect onincreasing the number of stops before returning to the key locale than the treatment with FLX10 alone. After 40 days, PUR10 treatment enhanced the number of stops in OCD-like rats compared to the PUR5 treatment (Figure 3d).

#### 3.2.2. Decreased Depression-Like Behavior after Purmorphamine Treatment

**Figure 4:**
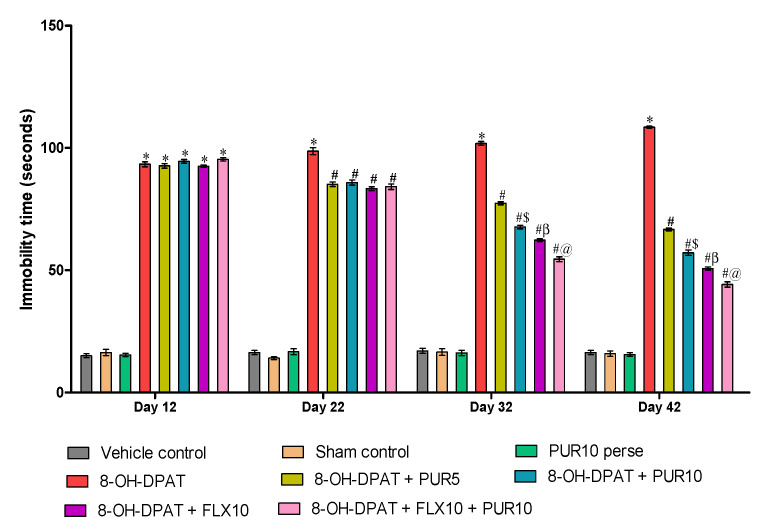
Effect of purmorphamine on immobility time in 8-OH OCD. Statistical analysis followed by two-way ANOVA (post-hoc Bonferroni’s test). Values expressed as mean ± SEM (*n* = 6 rats per group). * *p* < 0.01 v/s vehicle control, sham control and PUR10 per se; # *p* < 0.01 v/s 8-OH-DPAT; #$ *p* < 0.01 v/s 8-OH-DPAT + PUR5; #β *p* < 0.01 v/s 8-OH-DPAT + PUR5 and 8-OH-DPAT + PUR10; #@ *p* < 0.01 v/s 8-OH-DPAT + FLX10.

As shown in Figure 4, the results indicate a significant effect on immobility time in 8-OH OCD. Rats that were given 8-OH-DPAT regularly for one to seven days had considerably longer immobility times compared to the vehicle-control-, sham-control-, and PUR10-per se-treated rats. The immobility time decreased after PUR administration from day 8 to day 42, as observed by the treatment withthe antidepressant FLX10on day 22.

FLX10 administration, both alone and in combination with PUR10, resulted in a significant reduction in immobility time as (two-way ANOVA: F(21,120) = 160, *p* < 0.01) compared to the PUR5- and PUR10-treatment groups. Among the FLX-treated groups (FLX10 alone and along with PUR10), FLX10 and PUR10 have a more significant effect on reducing immobility time than the treatment withFLX10 alone in 8-OHOCD. Additionally, PUR10 significantly reduced the immobility time in the FST compared to the PUR5-treated rats in a dosage-dependent mannerafter 32 days. These results suggest the antidepressant effect of FLX and PUR when given alone and a more significant improvement in the antidepressant effect when given together in 8-OH OCD. 

#### 3.2.3. Decreased Marble-Burying Behavior after Purmorphamine Treatment

**Figure 5:**
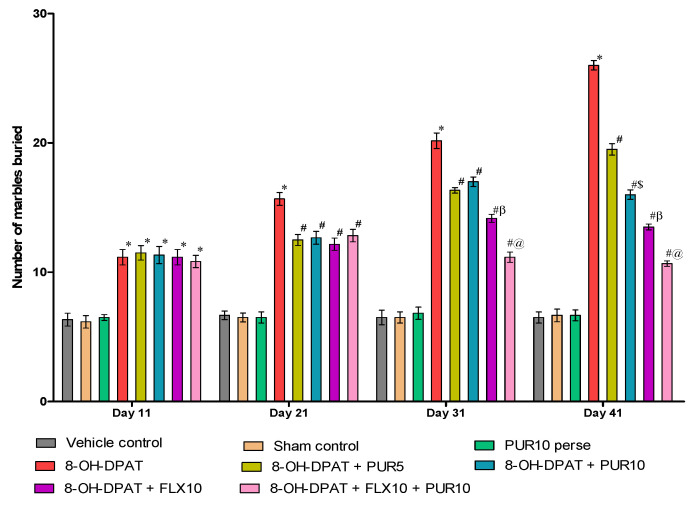
Effect of purmorphamine on marble-burying behavior in 8-OH OCD. Statistical analysis followed by two-way ANOVA (post-hoc Bonferroni’s test). Values expressed as mean ± SEM (*n* = 6 rats per group). * *p* < 0.01 v/s vehicle control, sham control and PUR10 per se; # *p* < 0.01 v/s 8-OH-DPAT; #$ *p* < 0.01 v/s 8-OH-DPAT + PUR5; #β *p* < 0.01 v/s 8-OH-DPAT + PUR5 and 8-OH-DPAT + PUR10; #@ *p* < 0.01 v/s 8-OH-DPAT + FLX10.

Figure 5 demonstrates the neuroprotective effect of PUR treatment on marble-burying behavior in 8-OH OCD, and a comparison with the standard OCD-treatment drug FLX. The results indicate that PUR and FLX significantly influenced marble-burying behavior in rats. The number of buried marbles increased significantly after seven days of continuous administration of 8-OH OCD compared to the vehicle control, sham control, and PUR10 per se. This increased the stereotyped and preservative behavior in rats. This stereotyped and repetitive marble-burying behavior was attenuated after FLX and PUR treatment from day 8 to 42 in 8-OH OCD.On day 31, FLX10, both alone and in combination with PUR10, exhibited a more substantial reduction in the number of buried marbles (two-way ANOVA F(21,120) = 28.04, *p* < 0.01) when compared to treatments with PUR alone at two different doses (5 mg/kg and 10 mg/kg). FLX in combination with PUR led to agreater reduction in the number of buried marbles compared to treatment withFLX10 alone. After 41 days, PUR10 showed a more significant decrease in buried marbles than PUR5 in a dosage-dependent manner.

#### 3.2.4. Decreased Spontaneous-Alternation-Behavior (SAB) Score after Purmorphamine Treatment

**Figure 6:**
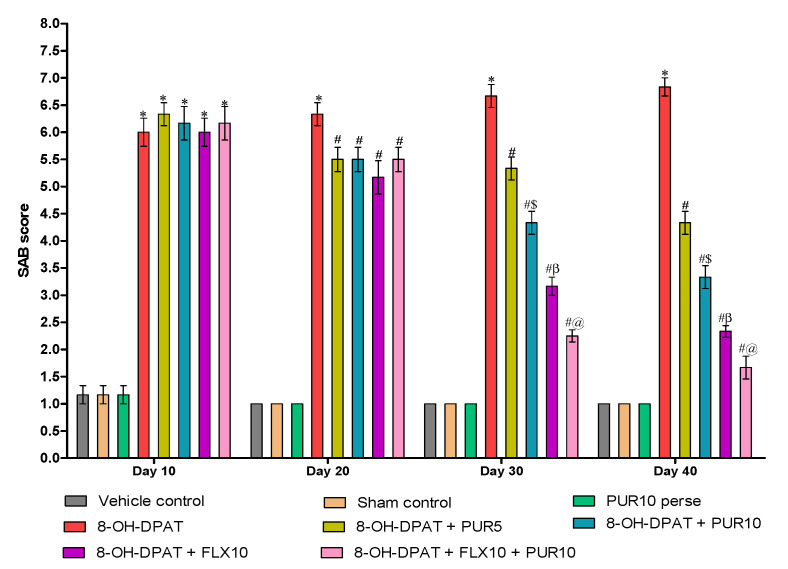
Effect of purmorphamine on spontaneous alternation behavior in 8-OH OCD. Statistical analysis followed by two-way ANOVA (post-hoc Bonferroni’s test). Values expressed as mean ± SEM (*n* = 6 rats per group). * *p* < 0.01 v/s vehicle control, sham control and PUR10 per se; # *p* < 0.01 v/s 8-OH-DPAT; #$ *p* < 0.01 v/s 8-OH-DPAT + PUR5; #β *p* < 0.01 v/s 8-OH-DPAT + PUR5 and 8-OH-DPAT + PUR10; #@ *p* < 0.01 v/s 8-OH-DPAT + FLX10.

SAB alterations are pharmacological paradigms that are commonly utilized to research anti-OCD properties. The chronic administration of 8-OH-DPAT for seven days in Wistar rats significantly increased the SAB score compared to the vehicle-control-, sham control-, and PUR10-per se-group rats. Regular oral SSRI treatment, i.e., FLX, from day 8 to day 42 significantly improved the SAB score at a 10 mg/kg dose compared to the PUR-treatment groups. However, co-administration of FLX andPUR at a dose of 10 mg/kg resulted in a more significant reduction in the SAB score (two-way ANOVA: F(21,120) = 25.14, *p* < 0.01)at day 30 than FLX10 treatment alone in 8-OH OCD.Treatment with PUR alone, as withFLX, reverses the 8-OH-DPAT-induced increase in the SAB score in a dose-dependent manner. On day 40 of the protocol schedule, PUR10 significantly reduced the SAB score compared to PUR5 (Figure 6).

#### 3.2.5. Reduction in Excessive Lever-Pressing Behavior after Purmorphamine Treatment

**Figure 7:**
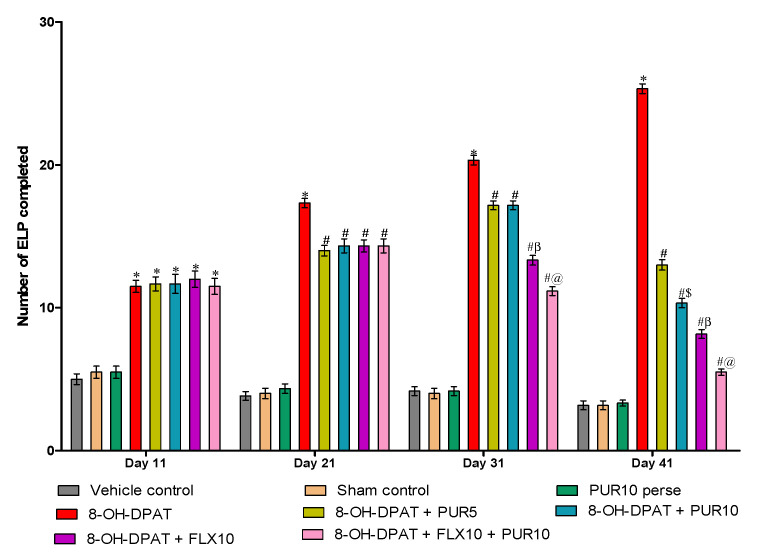
Effect of purmorphamine on excessive-lever-pressing behavior in 8-OH OCD. Statistical analysis followed by two-way ANOVA (post-hoc Bonferroni’s test). Values expressed as mean ± SEM (*n* = 6 rats per group). * *p* < 0.01 v/s vehicle control, sham control and PUR10 per se; # *p* < 0.01 v/s 8-OH-DPAT; #$ *p* < 0.01 v/s 8-OH-DPAT + PUR5; #β *p* < 0.01 v/s 8-OH-DPAT + PUR5 and 8-OH-DPAT + PUR10; #@ *p* < 0.01 v/s 8-OH-DPAT + FLX10.

The continuous excessive lever presses completed (ELP-C) signifies the compulsive and preservative behavior. Figure 7 represents the number of ELP-C in various treatment groups. The ELP-C considerably increased after a continuous seven-day injection of 8-OH-DPAT from day one to day seven compared to the vehicle-control, sham-control, and PUR-per se groups. Following that, PUR and FLX administration from day 8 to 42 had a significant anti-compulsive effect by lowering the ELP-C. In 8-OH OCD, the co-administration of FLX10 and PUR10 results in a more significant reduction in the ELP-C (two-way ANOVA: F(21,120) = 54.63, *p* < 0.01)thanoral treatment with only FLX10 after 30 days. However, FLX10 treatment ofOCD-like rats lowered the ELP-C more than the PUR5 and PUR10 treatments in OCD-like rats. Furthermore, PUR10 demonstrated a dose-dependent anti-compulsive effect and was more effective than PUR5 atreducing the ELP-C in 8-OH OCD on day 41.

### 3.3. Effect of Purmorphamineon Neurochemical Changes in 8-OH OCD

#### 3.3.1. Increased Smo-Shh Level after Purmorphamine Treatment

The chronic treatment withneurotoxin 8-OH-DPAT in Wistar rats demonstrated a substantial decrease in the level of Smo-Shh in the rats’ brain-homogenate, blood-plasma, and CSF samples compared to the vehicle-control, sham-control, and PUR10-per se groups. The groups that were treated with a co-administration of FLX10 and PUR10 FLX10 were found to have more significantly increased Smo-Shh levelsin their brain homogenate (one-way ANOVA:F(7,35) = 1.507, *p* < 0.01), blood plasma (one-way ANOVA: F(7,35) = 0.260, *p* < 0.01) and CSF (one-way ANOVA: F(7,35) = 1.615, *p* < 0.01) as compared to the PUR5- and PUR10-treated groups. Additionally, compared to treatment with FLX10 alone, treatment with FLX10 and PUR10 combined had a more significant restoration of the reducedSmo-Shh level in 8-OH OCD. Furthermore, compared to PUR5, PUR10 treatment restored the lower levels of Smo-Shh in OCD like rats’ brain homogenate, blood plasma, and CSF. These results indicate that the level of Smo-Shh decreased in the OCD-induced experimental rats, then increased after the treatment with purmorphamine and the standard treatment of fluvoxamine (Table 1).

#### 3.3.2. Decreased Apoptotic Markers Level after Purmorphamine Treatment

After seven days of 8-OH-DPAT administration, the levels of apoptotic markers such as Bax and Caspase-3 in the blood plasma and brain homogenate were relatively high compared to the vehicle-control, sham-control and PUR10-per se groups. In addition, the level of the anti-apoptotic marker Bcl-2 was shown to be significantly lower compared to the vehicle-control, sham-control, and PUR10-per se groups.This increase in the level of Caspase-3 (one-way ANOVA: F(7,35) = 1.783, *p* < 0.01) and Bax (one-way ANOVA: F(7,35) = 0.189, *p* < 0.01) and decrease in the level of Bcl-2 (one-way ANOVA: F(7,35) = 1.989, *p* < 0.01) was restored after FLX and PUR treatment from day 8th to 42nd in 8-OH-DPAT-induced experimental model of OCD in adult rats. 

FLX10 mg/kg administration, both alone and in combination with PUR10 mg/kg, resulted in a more significant decrease in Caspase-3 and Bax and an increase in Bcl-2 compared to the PUR5 mg/kg and PUR10 mg/kg treatment groups. FLX10 mg/kg combination with PUR10 mg/kg treated groups had a more significant effect in reducing the level of caspase-3 and Bax and increased level of Bcl-2 compared with FLX10 mg/kg alone treated OCD rat groups. PUR10 mg/kg has a more considerable effect on brain homogenate levels of caspase-3, Bax, and attenuation of decreased Bcl-2 in a dose-dependent manner than PUR5 mg/kg. The same significance was observed for Caspase-3 (one-way ANOVA: F(7,35) = 1.297, *p* < 0.01), Bax(one-way ANOVA: F(7,35) = 0.863, *p* < 0.01) and Bcl-2 (one-way ANOVA: F(7,35) = 1.422, *p* < 0.01) levels in blood plasma among different groups. This reveals increased apoptosis in 8-OH-DPAT-induced OCD-like rats, reduced by purmorphamine and fluvoxamine administration, indicating anti-apoptotic activity (Table 2).

#### 3.3.3. Decreased Inflammatory Cytokines Level after Purmorphamine Treatment

TNF-α-level evaluation by ELISAshowed that the repetitive intra-dorsal raphe-nucleus injection of 8-OH-DPAT triggers the release of TNF-α in the blood plasma and brain homogenate compared to the vehicle-control, normal-control, and PUR10-per se groups. The rat groups treated with FLX and PUR hadsignificantly decreased levels of TNF-α (one-way ANOVA: F(7,35) = 0.315, *p* < 0.01)and IL-1β (one-way ANOVA: F(7,35) = 2.254, *p* < 0.01)after 42 days of treatment. Among these treatment groups, those treated with FLX10, both alone and in combination with PUR10, indicate a more significant potential benefit in lowering the increased levels of pro-inflammatory cytokines than the PUR5- and PUR10-treated groups. Additionally, FLX10 and PUR10 treatment showeda considerably more reduced level of inflammatory markers than treatment with FLX10 alone in OCD rat groups. 

PUR10-treated OCD-like rats had lower levels of TNF-α and IL-1β in the brain homogenate than the PUR5-group rats, indicating a dose-dependent effect. The same significance was observed for TNF-α(one-way ANOVA: F(7,35) = 1.773, *p* < 0.01)and IL-1β (one-way ANOVA: F(7,35) = 0.756, *p* < 0.01)levels in the blood plasma among different groups. This study indicates that the intra-dorsal raphe-nucleus injection of 8-OH-DPAT causes an immunological response and the release of inflammatory cytokines in the rat brain, which is suppressed by purmorphamine treatment, which is similar to fluvoxamine treatment (Table 3).

#### 3.3.4. Restoration of Neurotransmitters Level after Purmorphamine Treatment

In the experimental study, serotonin and dopamine levels were shown to be lower in the brain homogenate of 8-OH-DPAT-treated rats; however, glutamate levels were found to be higher when compared to the vehicle-control, sham-control, and PUR10-per se groups. After 42 days, this neurotransmitter imbalance began to be restored in the FLX- and PUR-treated groups. FLX10, alone or in combination with PUR10, has a greater potential effect on raising serotonin (one-way ANOVA: F(7,35) = 0.536, *p* < 0.01) and dopamine (one-way ANOVA: F(7,35) = 0.415, *p* < 0.01) while decreasing glutamate levels (one-way ANOVA: F(7,35) = 0.417, *p* < 0.01) than the PUR5- and PUR10-treated groups. 

Compared tothe groups treated with FLX10 alone, FLX10 in combination with PUR10 had a more significant effect onrestoring neurotransmitter levels in the brain homogenate in8-OH OCD. Additionally, PUR10 treatment resulted in a significant increase in serotonin and dopamine and a decrease in glutamate in the brain homogenate of PUR10-treated rats compared to the PUR5-treated groups. These findings show that lower levels of serotonin and dopamine and elevated levels of glutamate cause an increase in repetitive and compulsive behavior in OCD-like rats, which is alleviated following a 42-day administration with PUR and the standard medication FLX (Table 4).

#### 3.3.5. Decreased Oxidative-Stress-Marker Levelsafter Purmorphamine Treatment

At the end of the experiment, the level of oxidative-stress markers was measured in the rats’ brain homogenate. Compared to the vehicle-control, sham-control, and PUR10-per se groups, the 8-OH-DPAT-treated groups had higher AChE, MDA, LDH, and nitrite levels and lower levels of reduced GSH and SOD. After PUR and FLX treatment from day 8 to day 42, the levels of these oxidative-stress markers becomerestored. FLX10treatment (alone and in combination with PUR10) showed a more significant effect ondecreasing the level of AChE (one-way ANOVA: F(7,35) = 0.615, *p* < 0.01), MDA (one-way ANOVA: F(7,35) = 0.937, *p* < 0.01), LDH (one-way ANOVA: F(7,35) = 0.662, *p* < 0.01) and nitrite (one-way ANOVA: F(7,35) = 0.463, *p* < 0.01) as compared to the PUR5- and PUR10-treatment groups. 

Additionally, FLX10 treatment alone and in combination with PUR10 showed a significant effect onincreasing GSH and SOD than PUR5 and PUR10treatment. Compared to the groups treated with FLX10 alone, FLX10 in combination with PUR10 had a more significant effect onrestoring oxidative-stress-marker levels in the brain homogenate in 8-OHOCD. PUR10 treatment showed a more significant decrease in AChE, MDA, LDH, and nitrite and increased the amount of decreased GSH and SOD in the brain homogenate compared to thePUR5-treated OCD-rat groups in a dose-dependent manner. This study found that FLX and theSmo-Shh agonist PUR protects against oxidative stress in 8-OH OCD (Table 5).

## 4. Discussion

8-OH-DPATinduced compulsive checking behavior in rats, whereby rats were unable to resist returning to a key locale during exploration of the open field [77]. This repetitive exploration was due to their compulsive behavior, in which they returned to the key locale again and again [55]. Our study found that direct injections of 8-OH-DPAT into the dorsal raphe nucleus for seven days induced compulsive checking behavior in rats by fulfilling all four criteria measures. After long-term PUR treatment of8-OH OCD, this compulsive behavior was improved, similar to FLX treatment with less potency. However, the combination of PUR and FLX both impart more significant improvements in compulsive behavior than the treatment with PUR or FLX alone.

Spontaneous alternation behavior in the T-maze is considered an appropriate model of OCD for assessing preservative behavior [16,78,79]. The 5-HT1A-receptor activator 8-OH-DPAT has been observed to induce repeated consecutive entries into the same arm, also known as preservation, which mimics the repetitive behavior of OCD patients [80,81].

In the current findings, PUR’s anti-compulsive effect was measured by its potential to prevent 8-OH-DPAT-induced impairment of spontaneous alternation behavior compared to the standard OCD treatment fluvoxamine. PUR5 and PUR10 were also found to be effective in restoring alternation behavior but had a less-significant effect when compared with FLX.

Marble-burying behavior was evaluated to measure characteristic repetitive compulsive-like behavior in OCD-like rats. It is a rodent’s defensive and unconditional behavior in which they repeatedly bury marbles that are not associated with any physical harm [57]. During this activity, they do not become habituated after repeated testing and continue to perform the process. Mice’s marble-burying behavior shave been used as an animal model to test anti-OCD medications [81,82]. In our study, rats were used as experimental animals, and OCD-like symptoms were developed by injecting 8-OH-DPAT into the dorsal raphe nucleus, resulting in excessive marble burying and anxious behavior. Previously, studies were conducted to assess marble-burying behavior in rats [83,84,85]. After FLX10, PUR5 and PUR10 therapy, the in creasein the number of marbles buried due to8-OH-DPAT was reduced. These two medications (PUR and FLX) worked better together than they did separately.

Signal attenuation is a model of OCD in which the rat excessively presses the lever not to collect the reward after signal-attenuation training [60]. This behavior is similar to the unusual repetitive behavior of OCD patients. The clinically utilized anti-OCD medicine FLX decreased the 8-OH-DPAT-induced repetitive behavior. PUR administration at two different dosages (5 and 10 mg/kg) was also found to have a comparable impact in reducing the number of excessive lever presses completed (ELP-C) in a dose-dependent manner.

A forced-swim test was performed to assess the depressive effect in OCD as SSRIs are the principal therapeutic agent used in antidepressants [86]. The depressive effect was measured using immobility time, the length of time the rat remains immobile in the water without moving both limbs. In 8-OH OCD, there was a significant change in immobility time, which was alleviated by PUR treatment, similar to FLX.

Neurochemical changes such as neurotransmitter imbalance, apoptosis, oxidative damage, and inflammation of neuronal cells are significant causes of the onset of compulsive-like behavior [4,87]. While there is no direct evidence for the role of the Smo-Shh agonist PUR in treating OCD, it can show a protective effect. Therefore, in this investigation, we assessed the neurochemical alterations and the protective effect of PUR in treating and restoring these changes in 8-OH OCD.

OCD is primarily defined by abnormalities in the levels of various neurotransmitters in the brain. The most widely involved neurotransmitter in OCD is serotonin, which regulates various behavioral functions. Many clinical studies on OCD patients have also reported the significant effect of serotonin-reuptake inhibitors in treating OCD [88,89,90,91].

Serotonin and dopamine deficiency impairs the motivational circuit related to the CSTC pathway and regulates behavioral characteristics [92,93,94,95,96]. Glutamate was reduced and serotonin was elevated by 8-OH-DPAT in this study, resulting in repetitive and compulsive behavior. This imbalance was dose-dependently restored by the Smo-Shh agonist PUR and the standard medication FLX.

Our study found that the level of Smo-Shh was reduced after seven days of continuous injection with 8-OH-DPAT. This reduced level of Smo-Shh leads to increased neuroinflammation, oxidative stress, and apoptosis [42]. Thus, PUR treatment significantly restores Smo-Shh and protects the neuronal cells against damage and neurochemical alterations. Neuroinflammation and apoptosis play a significant role in OCD where apoptotic markers, IL-1β, and TNF-ά are important factors of these processes. Apoptotic and cytokine levels have been observed to be elevated in people with obsessive-compulsive disorder (OCD) [97]. These increased apoptotic and inflammatory-cytokine levels leading to cytotoxic damage affect the neuronal transmission and increase the severity of OCD. The statements above support our findings that an increase in inflammatory and apoptotic markers following 8-OH-DPAT was later decreased by PUR, demonstrating its anti-inflammatory and anti-apoptotic effect [42]. PUR has been shown to be an anti-inflammatory and antioxidant in various CNS diseases, including Parkinson’s disease (PD), post-stroke depression (PSD), and autism [41,46,98]. 

Oxidative stress is closely linked with anxiety and depression [99]. This indicates that oxidative damage plays a major role in OCD pathogenesis [100]. The combination of PUR and FLX was also evaluated in order to examine the combined effect of these drugs on oxidative-stress markers. Additionally, the level of antioxidants significantly increased after long-term PUR treatment, signifying its protective effect against oxidative damage to neurons.

In summary, the current investigation found that PUR had a neuroprotective impact in alleviating 8-OH-DPAT-induced behavioral and neurochemical changes in OCD. These favorable benefits of PUR in attenuating behavioral alterations may be related to its ability to restore neurotransmitter levels and prevent brain damage via its anti-inflammatory and antioxidant characteristics. The considerable effect of PUR on OCD-like rats, when given in conjunction with FLX, suggests that it can be employed as a combination therapy in treating OCD more quickly and effectively. It is the first study to show that the Smo-Shh agonist PUR has a neuroprotective effect in OCD. Despite the lack of evidence regarding purmorphamine’s safety in humans, this chemical should be investigated further for the treatment of neurotoxicity linked with various infections, such as COVID-19.

**Limitations:** In order to validate and confirm the mechanistic process behind the protective effect of PUR on treating OCD, various knock-in and knock-out studies of Smo-Shh genes are required. The primary drawback of this study is the use of whole-brain homogenate for neurochemical analysis and the absence of immunohistology and gross-pathology studies regarding the area-specific molecular mechanistic action of PUR. Along with this, gross-pathological, immunohistological, and interaction studies between FLX and PUR need to be performed in order to strengthen these studies’ findings and support the rationale for the clinical use of PUR, both alone and in combination with FLX, in preventing and managing OCD. Despite some limitations, this study signifies that the evaluation of serotonin, Smo-Shh in OCD patients, can be a possible biomarker in identifying OCD and can be prevented by early detection.

## 5. Conclusions

Finally, this study found that purmorphamine has a neuroprotective effect in adult rats against the 8-OH-DPAT-induced experimental model of OCD. This is the first study to demonstrate that purmorphamine has a significant neuroprotective effect as a prospective medication for treating and managing OCD-like neurobehavioral abnormalities. The results of this study indicate that the Smo-Shh and serotonin assessments can be a possible candidate for the treatment of OCD-related neurocomplications.

## Figures and Tables

**Table 1:** Effect of purmorphamine on the restoration of Smo-Shh level in 8-OH OCD.

S.no.	Groups	Smo-Shh Protein Level		
		Brain Homogenate (nM/µg Protein)	Blood Plasma (ng/mL)	CSF (ng/mL)
1.	Vehicle control	11.89 ± 0.223	5.17 ± 0.030	2.49 ± 0.007
2.	Sham control	11.65 ± 0.191	5.11 ± 0.019	2.51 ± 0.009
3.	PUR10 per se	11.78 ± 0.187	5.12 ± 0.015	2.50 ± 0.009
4.	8-OH-DPAT	3.79 ± 0.102 *	2.73 ± 0.054 *	1.21 ± 0.011 *
5.	8-OH-DPAT + PUR5	4.78 ± 0.055 ^#^	3.23 ± 0.017 ^#^	1.46 ± 0.007 ^#^
6.	8-OH-DPAT + PUR10	6.00 ± 0.070 ^#$^	3.83 ± 0.024 ^#$^	1.76 ± 0.007 ^#$^
7.	8-OH-DPAT + FLX10	7.63 ± 0.105 ^#β^	4.16 ± 0.011 ^#β^	2.06 ± 0.010 ^#β^
8.	8-OH-DPAT + PUR10 + FLX10	9.05 ± 0.095 ^#@^	4.65 ± 0.017 ^#@^	2.25 ± 0.007 ^#@^

Statistical analysis followed by one-way ANOVA (post-hoc Tukey’s test). Values expressed as mean ± SEM (*n* = 6 rats per group).* *p* < 0.01 v/s vehicle control, sham control and PUR10 per se; ^#^ *p* < 0.01 v/s 8-OH-DPAT; ^#$^ *p* < 0.01 v/s 8-OH-DPAT + PUR5; ^#@^ 8-OH-DPAT + FLX10; ^#β^ *p* < 0.01 v/s 8-OH-DPAT + PUR5 and 8-OH-DPAT + PUR10.

**Table 2:** Effect of purmorphamine on Caspase-3, Bax, and Bcl-2 levels in 8-OH OCD.

S. no.	Groups	Apoptotic Markers					
		Caspase-3		Bax		Bcl-2	
		Brain Homogenate (nM/mg Protein)	Blood Plasma (ng/mL)	Brain Homogenate (ng/mg Protein)	Blood Plasma (ng/mL)	Brain Homogenate (ng/mg Protein)	Blood Plasma (ng/mL)
1.	Vehicle control	110.80 ± 0.691	1.66 ± 0.008	7.27 ± 0.071	1.15 ± 0.007	38.06 ± 0.304	9.78 ± 0.010
2.	Sham control	110.70 ± 0.559	1.67 ± 0.008	7.30 ± 0.089	1.15 ± 0.007	38.12 ± 0.352	9.79 ± 0.010
3.	PUR10 per se	110.70 ± 0.792	1.67 ± 0.010	7.37 ± 0.075	1.15 ± 0.007	38.05 ± 0.390	9.79 ± 0.011
4.	8-OH-DPAT	160.90 ± 0.939 *	6.28 ± 0.006 *	14.53 ± 0.180 *	5.68 ± 0.007 *	24.30 ± 0.346 *	3.59 ± 0.007 *
5.	8-OH-DPAT + PUR5	151.70 ± 0.581 ^#^	5.57 ± 0.009 ^#^	13.47 ± 0.043 ^#^	4.77 ± 0.007 ^#^	27.03 ± 0.231 ^#^	4.43 ± 0.007 ^#^
6.	8-OH-DPAT + PUR10	141.00 ± 0.546 ^#$^	4.45 ± 0.011 ^#$^	11.89 ± 0.033 ^#$^	3.56 ± 0.007 ^#$^	29.06 ± 0.120 ^#$^	5.64 ± 0.009 ^#$^
7.	8-OH-DPAT + FLX10	132.70 ± 0.365	3.36 ± 0.007 ^#β^	10.49 ± 0.064 ^#β^	2.86 ± 0.007 ^#β^	33.04 ± 0.204 ^#β^	6.51 ± 0.007 ^#β^
8.	8-OH-DPAT + PUR10 + FLX10	123.20 ± 0.345 ^#@^	2.21 ± 0.007 ^#@^	9.41 ± 0.027 ^#@^	1.92 ± 0.007 ^#@^	35.70 ± 0.103 ^#@^	7.87 ± 0.007 ^#@^

Statistical analysis followed by one-way ANOVA (post-hoc Tukey’s test). Values expressed as mean ± SEM (*n* = 6 rats per group). * *p* < 0.01 v/s vehicle control, sham control and PUR10 per se; ^#^ *p* < 0.01 v/s 8-OH-DPAT; ^#$^ *p* < 0.01 v/s 8-OH-DPAT + PUR5; ^#β^ *p* < 0.01 v/s 8-OH-DPAT + PUR5 and 8-OH-DPAT + PUR10; ^#@^ *p* < 0.01 v/s 8-OH-DPAT + FLX10.

**Table 3:** Effect of purmorphamine on TNF-α and IL-1β level in 8-OH OCD.

S. no.	Groups	Cytokine Level			
		TNF-α		IL-1β	
		Brain Homogenate (pg/mg Protein)	Blood Plasma (ng/mL)	Brain Homogenate (pg/mg Protein)	Blood Plasma (ng/mL)
1.	Vehicle control	35.42 ± 0.242	4.49 ± 0.014	18.75 ± 0.126	14.47 ± 0.007
2.	Sham control	35.80 ± 0.278	4.50 ± 0.014	18.92 ± 0.318	14.47 ± 0.010
3.	PUR10 per se	35.53 ± 0.213	4.49 ± 0.011	18.67 ± 0.291	14.48 ± 0.007
4.	8-OH-DPAT	76.44 ± 0.725 *	9.49 ± 0.009 *	33.22 ± 0.198 *	66.48 ± 0.008 *
5.	8-OH-DPAT + PUR5	67.40 ± 0.657 ^#^	8.47 ± 0.006 ^#^	28.35 ± 0.411 ^#^	55.39 ± 0.012 ^#^
6.	8-OH-DPAT + PUR10	58.30 ± 0.221 ^#$^	7.52 ± 0.009 ^#$^	25.78 ± 0.223 ^#$^	43.71 ± 0.014 ^#$^
7.	8-OH-DPAT + FLX10	50.01 ± 0.206 ^#β^	6.77 ± 0.009 ^#β^	23.08 ± 0.276 ^#β^	37.48 ± 0.014 ^#β^
8.	8-OH-DPAT + PUR10 + FLX10	42.05 ± 0.170 ^#@^	5.37 ± 0.009 ^#@^	21.22 ± 0.279 ^#@^	24.95 ± 0.008 ^#@^

Statistical analysis followed by one-way ANOVA (post-hoc Tukey’s test). Values expressed as mean ± SEM (*n* = 6 rats per group). * *p* < 0.01 v/s vehicle control, sham control and PUR10 per se; ^#^ *p* < 0.01 v/s 8-OH-DPAT; ^#$^ *p* < 0.01 v/s 8-OH-DPAT + PUR5; ^#β^ *p* < 0.01 v/s 8-OH-DPAT + PUR5 and 8-OH-DPAT + PUR10; ^#@^ *p* < 0.01 v/s 8-OH-DPAT + FLX10.

**Table 4:** Effect of purmorphamine on neurotransmitter levels in the brain samples of 8-OH OCD.

S. no.	Groups	Neurotransmitters		
		Serotonin (ng/mg Protein)	Glutamate (ng/mg Protein)	Dopamine (ng/mg Protein)
1.	Vehicle control	44.24 ± 0.191	114.40 ± 0.208	95.42 ± 0.294
2.	Sham control	44.26 ± 0.205	113.90 ± 0.333	95.74 ± 0.337
3.	PUR10 per se	44.06 ± 0.197	113.90 ± 0.307	95.50 ± 0.335
4.	8-OH-DPAT	16.33 ± 0.290 *	305.80 ± 0.316 *	31.48 ± 0.394 *
5.	8-OH-DPAT + PUR5	22.34 ± 0.232 ^#^	214.30 ± 0.260 ^#^	43.10 ± 0.222 ^#^
6.	8-OH-DPAT + PUR10	27.37 ± 0.223 ^#$^	186.60 ± 0.248 ^#$^	52.00 ± 0.392 ^#$^
7.	8-OH-DPAT + FLX10	32.87 ± 0.186 ^#β^	167.50 ± 0.639 ^#β^	63.05 ± 0.453 ^#β^
8.	8-OH-DPAT + PUR10 + FLX10	38.23 ± 0.236 ^#@^	142.20 ± 0.299 ^#@^	74.19 ± 0.448 ^#@^

Statistical analysis followed by one-way ANOVA (post-hoc Tukey’s test). Values expressed as mean ± SEM (*n* = 6 rats per group). * *p* < 0.01 v/s vehicle control, sham control and PUR10 per se; ^#^ *p* < 0.01 v/s 8-OH-DPAT; ^#$^ *p* < 0.01 v/s 8-OH-DPAT + PUR5; ^#β^ *p* < 0.01 v/s 8-OH-DPAT + PUR5 and 8-OH-DPAT + PUR10; ^#@^ *p* < 0.01 v/s 8-OH-DPAT + FLX10.

**Table 5:** Effect of purmorphamine on oxidative-stress-marker levels in 8-OH OCD.

S.no.	Groups	Oxidative-Stress Markers					
		AChE (µM/mg Protein)	LDH (µM/mg Protein)	SOD (µM/mg Protein)	GSH (µM/mg Protein)	Nitrite (µM/mg Protein)	MDA (nM/mg Protein)
1.	Vehicle control	24.12 ± 0.133	117.70 ± 0.366	478.00 ± 0.684	36.51 ± 0.320	7.72 ± 0.216	35.39 ± 0.240
2.	Sham control	24.26 ± 0.208	117.20 ± 0.222	478.40 ± 0.546	36.64 ± 0.329	8.07 ± 0.186	36.20 ± 0.342
3.	PUR10 per se	24.42 ± 0.194	117.90 ± 0.329	478.20 ± 0.660	36.54 ± 0.352	7.91 ± 0.295	35.75 ± 0.418
4.	8-OH-DPAT	54.44 ± 0.354 *	401.90 ± 0.851 *	305.30 ± 0.646 *	11.61 ± 0.286 *	14.18 ± 0.259 *	76.24 ± 0.269 *
5.	8-OH-DPAT + PUR5	46.46 ± 0.337 ^#^	315.40 ± 1.042 ^#^	332.60 ± 0.595 ^#^	16.20 ± 0.171 ^#^	12.44 ± 0.169 ^#^	66.64 ± 0.362 ^#^
6.	8-OH-DPAT + PUR10	40.94 ± 0.226 ^#$^	275.70 ± 1.046 ^#$^	369.20 ± 0.460 ^#$^	22.71 ± 0.342 ^#$^	11.40 ± 0.091 ^#$^	59.21 ± 0.383 ^#$^
7.	8-OH-DPAT + FLX10	35.83 ± 0.444 ^#β^	195.80 ± 1.011 ^#β^	411.40 ± 0.642 ^#β^	28.11 ± 0.150 ^#β^	10.45 ± 0.131 ^#β^	51.38 ± 0.383 ^#β^
8.	8-OH-DPAT + PUR10 + FLX10	30.05 ± 0.212 ^#@^	145.30 ± 1.374 ^#@^	446.30 ± 0.534 ^#@^	32.45 ± 0.226 ^#@^	9.47 ± 0.092 ^#@^	43.55 ± 0.370 ^#@^

Statistical analysis followed by one-way ANOVA (post-hoc Tukey’s test). Values expressed as mean ± SEM (*n* = 6 rats per group). * *p* < 0.01 v/s vehicle control, sham control and PUR10 per se; ^#^ *p* < 0.01 v/s 8-OH-DPAT; ^#$^ *p* < 0.01 v/s 8-OH-DPAT + PUR5; ^#β^ *p* < 0.01 v/s 8-OH-DPAT + PUR5 and 8-OH-DPAT + PUR10; ^#@^ *p* < 0.01 v/s 8-OH-DPAT + FLX10.

## Data Availability

All data generated or analysed during this study are included in thisarticle. There are no separate or additional files.

## Abbreviations

TF’s	Transcriptional Factors
BBB	Blood Brain Barrier
PPA	Propionic Acid
5-HT	Serotonin
8-OH-DPAT	8-hydroxy-2-(di-n-propylamino) tetralin
8-OH OCD	8-hydroxy-2-(di-n-propylamino) tetralin treated rats with obsessive-compulsive disorder
AChE	Acetyl cholinesterase
CCB	Compulsive checking behavior
CNS	Central nervous system
CSF	Cerebrospinal fluid
CSTC	Corticostriatal-thalamocortical pathway
DA	Dopamine
DMSO	Dimethyl sulfoxide
FST	Forced Swim test
GSH	Glutathione
HPLC	High performance liquid chromatography
IDRN	Intra dorsal raphe nucleus
IL-1β	Interleukin-1β
LDH	Lactate dehydrogenase
MBB	Marble burying behavior
MDA	Malondialdehyde
OCD	Obsessive Compulsive disorder
PD	Parkinson’s disease
PUR	Purmorphamine
ROS	Reactive Oxidative species
SAB	Spontaneous alternation behavior
SHH	Sonic Hedgehog
SMO	Smoothened
SOD	Superoxide dismutase
SOD	Superoxide dismutase
SSRIs	Selective Serotonin Reuptake Inhibitors
TNF-α	Tumour necrosis factor
*v*/*v*	volume/volume
